# Anti-angiogenic action of hyperthermia by suppressing gene expression and production of tumour-derived vascular endothelial growth factor *in vivo* and *in vitro*

**DOI:** 10.1038/sj.bjc.6600268

**Published:** 2003-10-21

**Authors:** Y Sawaji, T Sato, A Takeuchi, M Hirata, A Ito

**Affiliations:** Department of Biochemistry, School of Pharmacy, Tokyo University of Pharmacy and Life Science, 1432-1 Horinouchi, Hachioji, Tokyo 192-0392, Japan; Department of Thermotherapy, Luke Hospital, Nakano, Tokyo 165-0027, Japan

**Keywords:** VEGF, hyperthermia, angiogenesis, endothelial-cell, tumour metastasis

## Abstract

Vascular endothelial growth factor is an important angiogenic factor for tumour progression because it increases endothelial-cell proliferation and remodels extracellular matrix in blood vessels. We demonstrated that hyperthermia at 42°C, termed heat shock, suppressed the gene expression and production of vascular endothelial growth factor in human fibrosarcoma HT-1080 cells and inhibited its *in vitro* angiogenic action on human umbilical vein endothelial cells. The gene expression of alternative splicing variants for vascular endothelial growth factor, VEGF_121_, VEGF_165_ and VEGF_189_, was constitutively detected in HT-1080 cells, but the VEGF_189_ transcript was less abundant than VEGF_121_ and VEGF_165_. When HT-1080 cells were treated with heat shock at 42°C for 4 h and then maintained at 37°C for another 24 h, the gene expression of all vascular endothelial growth factor variants was suppressed. In addition, HT-1080 cells were found to produce abundant VEGF_165_, but much less VEGF_121_, both of which were inhibited by heat shock. Furthermore, the level of vascular endothelial growth factor in sera from six cancer patients was significantly diminished 2–3 weeks after completion of whole-body hyperthermia at 42°C (49.9±36.5 pg ml^−1^, *P*<0.01) as compared with that prior to the treatment (177.0±77.5 pg ml^−1^). On the other hand, HT-1080 cell-conditioned medium showed vascular endothelial growth factor-dependent cell proliferative activity and the augmentation of pro-matrix metalloproteinase-1 production in human umbilical vein endothelial cells. The augmentation of endothelial-cell proliferation and pro-matrix metalloproteinase-1 production was poor when human umbilical vein endothelial cells were treated with conditioned medium from heat-shocked HT-1080 cells. These results suggest that hyperthermia acts as an anti-angiogenic strategy by suppressing the expression of tumour-derived vascular endothelial growth factor production and thereby inhibiting endothelial-cell proliferation and extracellular matrix remodelling in blood vessels.

*British Journal of Cancer* (2002) **86**, 1597–1603. DOI: 10.1038/sj/bjc/6600268 www.bjcancer.com

© 2002 Cancer Research UK

## 

Angiogenesis is essential for tumour development and metastasis, and tumour-derived angiogenic factor(s) play(s) an important role in the formation of new vessels and tumour progression *in vivo* and *in vitro* (Weidner *et al*, 1991; Ferrara *et al*, 1992; Folkman, 1992). Vascular endothelial growth factor (VEGF) (Ferrara and Henzel, 1989; Leung *et al*, 1989), basic fibroblast growth factor (bFGF) (Abraham *et al*, 1986; Gospodarowicz *et al*, 1987), transforming growth factor-α (TGF-α) (Schreiber *et al*, 1986) and platelet-derived growth factor (Holmgren *et al*, 1991; Risau *et al*, 1992) all have been identified as angiogenic factors. Among them, VEGF is considered to be the pivotal factor in tumour neovascularisation, because it increases in endothelial-cell proliferation and migration (Leung *et al*, 1989; Connolly *et al*, 1989; Ferrara *et al*, 1992), enhancement of tumour growth *in vivo* (Kondo *et al*, 1993, 2000) and remodelling of perivascular matrices by augmenting proteinases such as matrix metalloproteinases (MMPs) (Fisher *et al*, 1994; Moses, 1997).

VEGF is a 34–42 kDa heparin-binding and dimeric glycoprotein, and four isoforms have been characterised. Three of these, VEGF_121_, VEGF_165_ and VEGF_189_ are composed of 121, 165 and 189 amino acids, respectively, and are generated by alternative splicing of eight exons (Tischer *et al*, 1991). VEGF_189_ is encoded by all the exons, and VEGF_165_ and VEGF_121_ are missing the amino acid residues corresponding to exon 6 and exons 6 and 7, respectively (Tischer *et al*, 1991). The fourth VEGF cDNA species, VEGF_206_ was discovered in a human foetal-liver cDNA library and the gene codes for a predicted protein with 206 amino acids. In comparison to VEGF_165_, VEGF_206_ contains an additional 41 amino acids between exons 5 and 7 as well as a basic 24 amino acid insertion also found in VEGF_189_ (Houck *et al*, 1991). VEGF_165_ and VEGF_121_ have been reported to be secreted in a wide variety of transformed cell lines (Senger *et al*, 1986; Connolly *et al*, 1989; Myoken *et al*, 1991; Kondo *et al*, 1994) and VEGF_165_ is an abundant species detectable in several tumours (Ferrara *et al*, 1992). In contrast, VEGF_189_ and VEGF_206_ exist on the cell surface as structural profiles possessing hydrophobic residues (Houck *et al*, 1991). On the other hand, several therapeutic studies show that the inhibition of the biological activity and/or the production of VEGF suppresses tumour angiogenesis and growth *in vivo* (Kim *et al*, 1993; Kondo *et al*, 1993; Asano *et al*, 1995; Borgstrom *et al*, 1996; Cheng *et al*, 1996; Saleh *et al*, 1996; Im *et al*, 1999), suggesting that tumour-derived VEGF plays a crucial role for tumour neovascularisation *in vivo*. Therefore, the suppression of VEGF expression in tumours would become a potent clinical strategy in cancer therapy for regulating tumour angiogenesis.

Hyperthermic treatment of malignant tumours is one cancer therapy by which the possible mechanism of the proliferation of tumour cells is relatively inhibited (Urano *et al*, 1983; Cellier *et al*, 1993). In addition, Toyota *et al* (1997) reported that a whole-body hyperthermia inhibits metastasis of breast cancer cells in rat *in vivo*. Moreover, it has been reported that not only progression but also metastasis of tumours are inhibited in most cases of cancer patients treated with a whole-body hyperthermia (Takeuchi *et al*, 1996, 1999). We recently reported that heat shock suppresses *in vitro* invasive activity of human fibrosarcoma HT-1080 cells by suppressing the production of membrane type 1-MMP (MT1-MMP) and the activation of proMMP-2/progelatinase A (Sato *et al*, 1999; Sawaji *et al*, 2000). On the other hand, Fajardo *et al* (1988) reported that hyperthermia inhibits angiogenesis *in vivo*. Therefore, it is likely that hyperthermia is efficacious for preventing tumour metastasis and invasion, but it remains unclear whether hyperthermia could influence the expression of VEGF in tumour cells.

In the present study, we investigated the effect of heat shock on the production of VEGF in HT-1080 cells and the biological activities of conditioned medium from heat-shocked HT-1080 cells for endothelial-cell proliferation and proMMP-1/interstitial procollagenase production. Heat shock suppressed the constitutively expressed gene and the production of alternative splicing variants of VEGF in HT-1080 cells. Similar suppression of VEGF level was observed *in vivo* when patients with different cancers were treated with the whole-body hyperthermia. In addition, the augmentation of endothelial-cell proliferation and proMMP-1 production was reduced in the conditioned medium from heat-shocked HT-1080 cells. Therefore, we suggest that hyperthermia suppresses angiogenesis by inhibiting the production of tumour-derived VEGF *in vivo* and *in vitro*.

## MATERIALS AND METHODS

### Cell culture and heat-shock treatment

Human fibrosarcoma HT-1080 cells (Health Science Research Bank, Osaka, Japan) were cultured in MEM (Life Technologies, Inc., Grand Island, NY, USA) supplemented with 10% FBS (Asahi Techno Glass Co., Tokyo, Japan) and MEM non-essential amino acids (Life Technologies). Human umbilical vein endothelial cells (HUVECs) (Takara Shuzo Co., Shiga, Japan) were cultured in EBM supplemented with 2% FBS, 10 ng ml^−1^ human epidermal growth factor and 12 μg ml^−1^ bovine brain extract (Takara Shuzo). In the heat-shock experiments, confluent HT-1080 cells were treated with heat shock at 42°C for 4 h in MEM supplemented with 0.2% lactalbumin hydrolysate and then incubated for another 24 h at 37°C (Sato *et al*, 1999; Sawaji *et al*, 2000). The harvested culture medium was used for assay of endothelial-cell proliferation and Western blot analysis as described below.

### Semiquantification of VEGF mRNA by reverse transcriptase-polymerase chain reaction (RT–PCR)

Cytoplasmic RNA in untreated and heat-shocked HT-1080 cells was isolated by ISOGEN (Nippon Gene Co., Toyama, Japan) according to the manufacturer's instructions. One microgram of the isolated RNA was subjected to the synthesis of first-strand cDNA by Moloney-murine leukaemia virus reverse transcriptase, RNase inhibitor (Roche Diagnostics, Tokyo, Japan) and oligo(dT)_12–18_ primer (Life Technologies) for 1 h at 37°C. One-tenth of the cDNA generated from the RT reaction was used for PCR amplification for human VEGF and human glyceraldehyde-3-phosphate dehydrogenase (GAPDH). To detect the individual splicing variants for VEGF, a common forward primer and variant-specific reverse primers were designed (Table 1

**Table 1:**
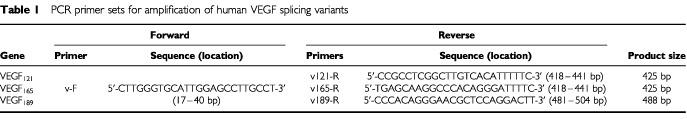
PCR primer sets for amplification of human VEGF splicing variants

). The forward and reverse primers for human GAPDH were 5′-CCACCCATGGCAATTCCATGGCA-3′ and 5′-TCTAGACGGCAGGTCAGGTCCACC-3′, respectively. Polymerase chain reaction (PCR) was performed with 92°C for 40 s, at 54°C for 40 s and 72°C for 1 min with 25–29 cycles for VEGFs and GAPDH. The amplified PCR products were analysed on 1% agarose gel and visualised by ethidium bromide staining. The PCR products were subcloned into pGEM-T vector (Promega, Madison, WI, USA), and then the cDNA sequence was confirmed with a Sequenase version 2.0 DNA sequencing kit (Amersham Biosciences, Tokyo, Japan) according to the manufacturer's instructions. The relative amounts of the amplified gene for VEGFs were quantified by densitometric scanning using the Image Analyzer LAS-1000 plus (Fuji Photo Film Co., Ltd., Tokyo, Japan) and then indicated after correction for that of GAPDH.

### Western blot analysis for VEGF and proMMP-1

The production of VEGF in HT-1080 cells, and that of proMMP-1 in HUVECs were monitored by Western blot analysis with antibodies against human VEGF and human proMMP-1, respectively. Briefly, the harvested culture medium was subjected to SDS–PAGE with 12.5% and 10% acrylamide gel to detect VEGF and proMMP-1, respectively, and then proteins separated in the gel were electrotransferred onto a nitrocellulose membrane as described previously (Takahashi *et al*, 1991). The membrane was reacted with rabbit anti-(human VEGF) antibody (IBL, Gunma, Japan) or sheep anti-(human proMMP-1) antibody (kindly provided by Dr Hideaki Nagase), which was then complexed with horseradish peroxidase-conjugated goat anti-(rabbit IgG)IgG or goat anti-(sheep IgG)IgG (Sigma Chemical Co., St. Louis, MO, USA), respectively. Immunoreactive VEGF and proMMP-1 were visualised with ECL-Western blotting detection reagents (Amersham Biosciences) according to the manufacturer's instructions. The relative amounts of VEGF and proMMP-1 were quantified by densitometric scanning using the Image Analyzer LAS-1000 plus (Fuji Photo Film).

### Endothelial-cell proliferation assay

HUVECs (500 cells well^−1^) were seeded into 96-multi well plates and cultured for 24 h at 37°C to achieve cell adhesion. The cells were washed twice with Ca^2+^- and Mg^2+^-free phosphate buffered saline (PBS) and then incubated for the indicated periods with the HT-1080 cell-conditioned medium which was diluted 1 : 1 (vol : vol) with 0.5% FBS/EBM. The proliferation of HUVECs was monitored by alamer Blue assay (Wako Pure Chemical Co., Osaka, Japan) (Ahmed *et al*, 1994) according to the manufacturer's instructions. The fluorescence was measured by excitation at 540 nm and emission at 590 nm.

### Neutralizing experiment with antibodies

The HT-1080 cell-conditioned medium was incubated first with 50 μg ml^−1^ of polyclonal antibody against human VEGF, human TGF-α, human tumour necrosis factor-α (TNF-α) (R&D Systems Inc., Minneapolis, MN, USA) or monoclonal antibody against human bFGF (Transduction Laboratories, Lexington, KY, USA) at 4°C for 24 h and then incubated with Protein A-Sepharose (Amersham Biosciences) for 1 h at room temperature. The Protein A-Sepharose-IgG complex was precipitated by centrifugation at 10 000×*g* for 10 min and the resultant supernatant was sterilised and then used for the assay of endothelial-cell proliferation and proMMP-1 production as described above.

### Whole-body hyperthermia

Whole-body hyperthermia was performed with a far-infrared radiation heat device, using the instruments RHD2002 and RHS7500 (Enthermics Medical Systems Inc., Menomonee Falls, WI, USA) (Robins *et al*, 1985). Patients were anaesthetised and then subjected to the hyperthermia by maintaining a temperature of 42–43°C locally in the hyperthermic chamber and 41.5–42°C systemically (rectal temperature) for 1 h (Takeuchi *et al*, 1996). The hyperthermic therapy was performed once a week for 4 weeks. Two–seven days prior to the first treatment, and 2–3 weeks after the completion of the whole-body hyperthermia, blood was collected from the patients and then the serum level of VEGF was measured by VEGF immunoassay. This therapeutic treatment was performed only after approval of the protocol by the ethics committee of the hospital and after obtaining informed consent.

### Measurement of VEGF

The levels of VEGF in sera from six cancer patients before and after whole-body hyperthermia were measured by human VEGF Immunoassay kit (R&D Systems) according to the manufacturer's instructions. A VEGF antibody contained in this kit recognises both VEGF_121_ and VEGF_165_, and thus the immunoassay can be used to determine mass values for human VEGF_121_ and VEGF_165_.

### Statistical analysis

Data were analysed by Student's *t*-tes*t*. *P*<0.01 was considered to be statistically significant.

## RESULTS

### Gene expression of VEGF variants in HT-1080 cells

**Figure 1:**
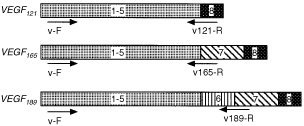
Structure of human VEGF mRNA. Exons are represented by box and numbered. Arrows indicate the specific primers for VEGF variants as shown in Table 1.

**Figure 2:**
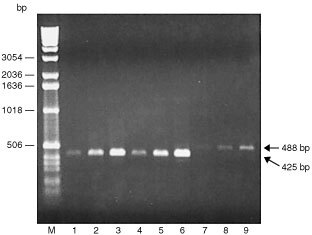
Characterisation of gene expression of VEGF splicing variants in human fibrosarcoma HT-1080 cells. Isolated RNA (1 μg) was subjected to RT–PCR analysis with 25 (lanes 1, 4 and 7), 27 (lanes 2, 5 and 8) and 29 cycles (lanes 3, 6 and 9) using specific primers for respective VEGF splicing variants; VEGF_121_, VEGF_165_ and VEGF_189_ as indicated in Figure 1 and Table 1. Two independent experiments were reproducible and typical data were shown. Lanes 1–3, VEGF_121_; lanes 4–6, VEGF_165_ and lanes 7–9, VEGF_189_.

The VEGF gene consists of the common exons 1–5 and the individually specific exons for the variants: exon 8 for VEGF_121_, exons 7 and 8 for VEGF_165_, and exons 6, 7 and 8 for VEGF_189_ (Figure 1

). A common forward primer for VEGF (v-F) (17–40 bp, exon 1) was designed downstream from the start codon (Table 1 and Figure 1). Specific reverse primers for VEGF_121_ (v121-R), VEGF_165_ (v165-R) and VEGF_189_ (v189-R) were also designed across exons 5 and 8 (418–441 bp), exons 5 and 7 (418–441 bp) and exons 6 and 7 (481–504 bp), respectively (Table 1 and Figure 1). We first characterised the expression of VEGF transcripts in HT-1080 cells by RT–PCR analysis using these PCR primers. As shown in Figure 2

, the gene expression of VEGF_121_ (425 bp), VEGF_165_ (425 bp) and VEGF_189_ (488 bp) was detected in HT-1080 cells and the amplification was cycle number-dependent. In addition, the gene level of VEGF_189_ was found to be less than that of VEGF_121_ and VEGF_165_ in HT-1080 cells. Furthermore, we confirmed that the DNA sequence of the amplified VEGF variants was completely identical to that in the previous paper by Leung *et al* (1989) (data not shown). However, we did not detect a VEGF_206_ transcript in HT-1080 cells by RT–PCR using the common forward primer and a specific reverse one that was designed with a terminal codon in exon 8 (data not shown).

### Heat shock suppresses gene expression and production of VEGF in HT-1080 cells

**Figure 3:**
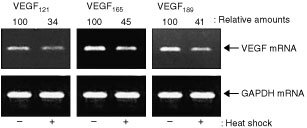
Heat shock suppresses gene expression of VEGF variants in HT-1080 cells. Confluent HT-1080 cells were treated with or without heat shock at 42°C for 4 h and then incubated for another 24 h. Isolated RNA was subjected to RT–PCR analysis with 27 cycles for VEGF_121_ and VEGF_165_ and with 29 cycles for VEGF_189_ as described in Figure 2. The relative amounts of VEGF mRNA were quantified by densitometric scanning followed by normalising against that of GAPDH mRNA and expressed taking the untreated HT-1080 cells as 100. Three independent experiments were reproducible and typical data were shown.

**Figure 4:**
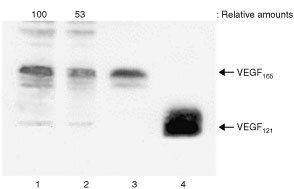
Heat shock suppresses production of VEGF in HT-1080 cells. Confluent HT-1080 cells were treated with or without heat shock as described in Figure 3. The harvested culture medium was subjected to Western blot analysis for VEGF as described in Materials and Methods. The relative amounts of VEGF_165_ were quantified by densitometric scanning and expressed taking the untreated HT-1080 cells as 100. Three independent experiments were reproducible and typical data were shown. Lane 1, untreated HT-1080 cells; lane 2, heat-shocked HT-1080 cells; lane 3, recombinant human VEGF_165_ (10 ng) and lane 4, recombinant human VEGF_121_ (20 ng).

We examined the influence of heat shock on the gene expression of VEGF variants in HT-1080 cells. When the cells were pretreated with heat shock at 42°C for 4 h and then incubated for another 24 h at 37°C, VEGF_121_, VEGF_165_ and VEGF_189_ transcripts were decreased to 34, 45 and 41%, respectively, of the values for the untreated cells (Figure 3

). Western blot analysis showed that HT-1080 cells produced abundant VEGF_165_ with the same mobility of recombinant human VEGF_165_ (Figure 4

, lane 1). The production of VEGF_165_ in heat-shocked HT-1080 cells was suppressed to 53% of the values for untreated cells (Figure 4, lane 2). In addition, VEGF_121_ was detected in HT-1080 cells, but in a much smaller amount than VEGF_165_, and its production was similarly suppressed by heat shock (Figure 4, lanes 1 and 2). Therefore, these results suggest that heat shock suppresses the production of VEGF_165_ and VEGF_121_ through the depression of their mRNA expression in HT-1080 cells.

### Effect of conditioned medium from heat-shocked HT-1080 cells on proliferation of HUVECs

**Figure 5:**
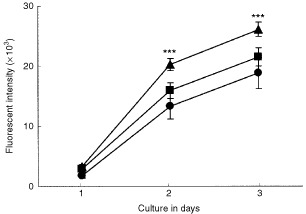
Proliferation of HUVECs by HT-1080 cell-derived conditioned medium. HUVECs (500 cells well^-1^) were treated with control medium (filled circles), with the HT-1080 cell-conditioned medium (filled triangles) or with the heat-shocked HT-1080 cell-conditioned medium (filled squares). The proliferation of HUVECs was monitored by alamer Blue assay as described in Materials and Methods. The data are the mean±s.d. of values from six wells at each point. ***Significantly different from HUVECs treated with control medium (*P*<0.001). Two independent experiments were reproducible and typical data were shown.

**Figure 6:**
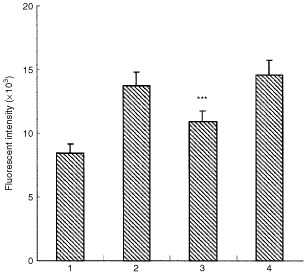
Characterisation of HT-1080 cell-derived factor for endothelial-cell proliferation. Conditioned medium from HT-1080 cells was pretreated with an antibody against VEGF (50 μg ml^−1^) or bFGF (50 μg ml^−1^) and then HUVECs (500 cells well^−1^) were treated with or without the conditioned medium. After 2 days treatment, the proliferation of HUVECs was monitored by alamer Blue assay as described in Figure 5. The data are the mean±s.d. of values from six wells. Two independent experiments were reproducible and typical data were shown. Lane 1, HUVECs cultured in control medium; lane 2, HUVECs treated with the HT-1080 cell-conditioned medium; lane 3, HUVECs treated with the HT-1080 cell-conditioned medium pretreated with VEGF antibody and lane 4, HUVECs treated with the HT-1080 cell-conditioned medium pretreated with bFGF antibody. ***Significantly different from HUVECs treated with the HT-1080 cell-conditioned medium (*P*<0.001).

VEGF possesses the mitogenic activity to cause proliferation of endothelial cells in angiogenesis (Leung *et al*, 1989; Connolly *et al*, 1989; Ferrara *et al*, 1992). We next examined the endothelial-cell proliferative activity of conditioned medium from untreated and heat-shocked HT-1080 cells. When the conditioned medium from HT-1080 cells was added to HUVECs, the endothelial-cell proliferation was increased in a time-dependent manner and doubled for 3 days (Figure 5

, filled triangles). However, the conditioned medium from heat-shocked HT-1080 cells no longer indicated proliferative activity toward HUVECs (Figure 5, filled squares). On the other hand, as shown in Figure 6

, the enhancement of endothelial-cell proliferation by the conditioned medium from HT-1080 cells (lane 2) was neutralised with an antibody against VEGF (lane 3), but not by an antibody to bFGF (lane 4), which is known as another angiogenic factor *in vivo* and *in vitro* (Abraham *et al*, 1986; Gospodarowicz *et al*, 1987). In addition, we confirmed that neither TGF-α nor TNF-α antibody interfered with the proliferative activity on HUVECs (data not shown). These results indicate that the enhancement of endothelial-cell proliferation by conditioned medium from HT-1080 cells was mediated specifically by VEGF, and suggest that heat shock is adequate for inhibiting the endothelial-cell proliferation by suppressing tumour-derived VEGF production.

### Effect of conditioned medium from heat-shocked HT-1080 cells on production of endothelial proMMP-1

**Figure 7:**
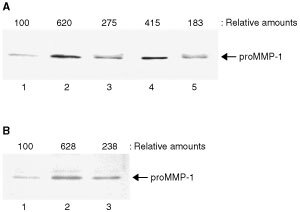
HT-1080 cell-derived VEGF stimulates HUVECs to produce proMMP-1. (**A**) Conditioned medium from HT-1080 cells and the culture medium supplemented with human recombinant VEGF_165_ (20 ng ml^−1^) were pretreated with or without an antibody against VEGF (50 μg ml^−1^) and then HUVECs were treated with these conditioned media for 24 h. The harvested culture medium was subjected to Western blot analysis for proMMP-1 as described in Materials and Methods. Lane 1, untreated HUVECs; lane 2, HUVECs treated with the HT-1080 cell-conditioned medium; lane 3, HUVECs treated with the HT-1080 cell-conditioned medium pretreated with VEGF antibody; lane 4, HUVECs treated with recombinant human VEGF_165_ and lane 5, HUVECs treated with recombinant human VEGF_165_ pretreated with VEGF antibody. (**B**) Confluent HUVECs were treated with or without conditioned medium from untreated or heat-shocked HT-1080 cells. Three independent experiments were reproducible and typical data were shown. Lane 1, untreated HUVECs; lane 2, HUVECs treated with the HT-1080 cell-conditioned medium and lane 3, HUVECs treated with the heat-shocked HT-1080 cell-conditioned medium. The relative amounts of proMMP-1 production were quantified by densitometric scanning and expressed taking the untreated HUVECs as 100.

The augmentation of proteolytic activity is crucial for extracellular matrix (ECM) remodelling in order to provide a permissive environment in which activated endothelial cells can proliferate and form new vessels (Moses, 1997; Liotta *et al*, 1991). In addition, endothelial MMPs such as MMP-1 and MT1-MMP have been shown to participate in ECM remodelling in the perivascular environment (Fisher *et al*, 1994; Hiraoka *et al*, 1998). We therefore investigated the effect of conditioned medium from HT-1080 cells on the production of proMMP-1 in HUVECs. Western blot analysis showed that the production of proMMP-1 in HUVEC was augmented by the conditioned medium from HT-1080 cells (6.2-fold) as well as by recombinant human VEGF_165_ (4.2-fold) (Figure 7A

, lanes 2 and 4, respectively). In addition, the augmentation of proMMP-1 production was inhibited by adding a neutralising antibody against VEGF (Figure 7A, lanes 3 and 5). Furthermore, the augmentation of proMMP-1 production was poor in HUVECs treated with the conditioned medium from heat-shocked HT-1080 cells rather than that from untreated HT-1080 cells (Figure 7B, lanes 2 and 3). These results suggest that heat shock is efficacious in the prevention of MMP-dependent ECM remodelling in the process of angiogenesis by suppressing the production of tumour-derived VEGF.

### Whole-body hyperthermia diminishes the serum level of VEGF in cancer patients *in vivo*

To clarify whether hyperthermia inhibits the production of VEGF *in vivo*, we investigated the serum level of VEGF in various cancer patients before and after whole-body hyperthermia at 42°C (Takeuchi *et al*, 1996, 1999). The patient characteristics are listed in Table 2

**Table 2:**
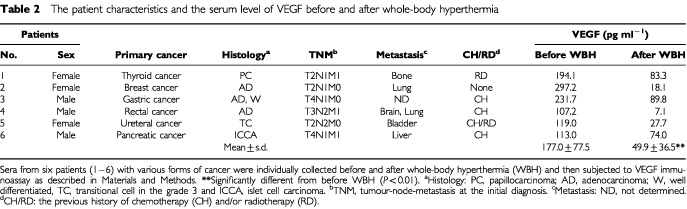
The patient characteristics and the serum level of VEGF before and after whole-body hyperthermia

. Six patients had various forms of advanced cancer, and the histology and tumour-node-metastasis (TNM) in the individual patients were characterised at the initial diagnosis. In addition, five of these patients had received previous chemotherapy and/or radiotherapy. When sera from these patients were collected 2–7 days prior to the first whole-body hyperthermia, the serum level of VEGF was higher than 100 pg ml^−1^ in all patients and its average (±s.d) was 177.0 (±77.5) pg ml^−1^ (Table 2). Two–three weeks after completion of the hyperthermic therapy, the level of VEGF decreased to 49.9 (±36.5) pg ml^−1^, which was almost the same as the normal VEGF level (Hyodo *et al*, 1998). Therefore, it is suggested that whole-body hyperthermia efficiently diminishes the level of VEGF in advanced cancer patients.

## DISCUSSION

VEGF has been characterised as existing in four isoforms; VEGF_121_, VEGF_165_, VEGF_189_ and VEGF_206_ (Ferrara *et al*, 1992). To individually and specifically amplify these variants by semi-quantitative RT–PCR, we designed unique PCR primers and demonstrated that HT-1080 cells expressed predominantly VEGF_121_ and VEGF_165_ mRNA as well as a smaller amount of the VEGF_189_ transcript, and did not express VEGF_206_. In addition, Western blot analysis showed that the immunoreactive VEGF was mostly VEGF_165_ and a smaller amount of VEGF_121_, while the mRNA level of both isoforms was the same in HT-1080 cells. No immunologically detected VEGF variants could be found in the cell-membrane fraction of HT-1080 cells (data not shown), although VEGF_189_ and VEGF_206_ exist as cell-associated forms (Houck *et al*, 1991). On the other hand, tumour cells have been shown to produce other angiogenic factors such as bFGF (Abraham *et al*, 1986; Gospodarowicz *et al*, 1987), TGF-α (Smith *et al*, 1987) and PDGF (Holmgren *et al*, 1991; Risau *et al*, 1992). We demonstrated that the proliferation of HUVECs by conditioned medium derived from HT-1080 cells was effectively prevented by adding VEGF antibody, but not by adding antibodies to bFGF, TGF-α and TNF-α (data not shown). Therefore, it is suggested that the angiogenic factor derived from HT-1080 cells is primarily VEGF_165_.

Exposing malignant cells to hyperthermia is a therapeutic strategy that prevents tumour progression by inhibiting the proliferation of tumour cells (Urano *et al*, 1983). Fajardo *et al* (1988) also reported that hyperthermia inhibits angiogenesis by interference with cell replication and/or inhibition of the migration of vascular endothelial cells. However, the effect of hyperthermia on the production of VEGF is not specified. In the present study, we demonstrated for the first time that heat shock suppresses the gene expression and the production of VEGF_165_ in HT-1080 cells. Moreover, the heat shock-mediated suppressions of both VEGF_165_ production and its gene expression were similarly observed in human squamous carcinoma A431 cells (data not shown). Thus, this novel evidence that heat shock directly down-regulates the expression of VEGFs in HT-1080 cells is likely to be ubiquitously observed in tumour cells.

Endothelial cells are basically quiescent, but when activated by VEGF, they turn into the angiogenic phenotype and then proliferate and migrate to form new vessels *in vivo* and *in vitro* (Weidner *et al*, 1991; Ferrara *et al*, 1992). In addition, VEGF-mediated neovascularisation is closely associated with tumour growth *in vivo* (Kim *et al*, 1993; Asano *et al*, 1995; Cheng *et al*, 1996; Im *et al*, 1999). In fact, Kim *et al* (1993) reported that the intraperitoneal injection of a specific monoclonal antibody for human VEGF_165_ inhibits the growth of rhabdomyosarcoma, glioblastoma multiforme and leiomyosarcoma cell lines in nude mice. Im *et al* (1999) also reported that an antisense cDNA molecule of VEGF induces anti-tumorigenic effects *in vivo* on human glioma tumours established in nude mice. Therefore, it is likely that VEGF is a clinical target molecule for cancer therapy. Thus, our finding that heat shock inhibited VEGF expression in tumour cells strongly suggests that hyperthermia might be one therapeutic strategy for preventing angiogenesis together with known therapeutic properties such as anti-tumorigenic activity *in vivo*.

Recently, Kanamori *et al* (1999) reported that hyperthermia at 44°C induces the expression of VEGF in SCC VII tumours in C3H/He mice, by the mechanism that heat-mediated vascular damage may attribute to hypoxia and thereafter tumour necrosis. This phenomenon is different from our finding, that heat shock suppresses VEGF expression in tumour cells. In our experiments, heat shock at 42°C for 4 h does not influence cellular functions such as biosynthesis of total proteins and tumour cell growth *in vitro* (Sato *et al*, 1999; Sawaji *et al*, 2000). Thus, the discrepancy may be due to the difference of experimental conditions such as temperature and heat-exposing period. Furthermore, we demonstrated that the level of VEGF in serum was diminished in all cancer patients treated with whole-body hyperthermia. Therefore, it is very likely that the *in vitro* findings in the present study reflect on the *in vivo* effect of whole-body hyperthermia, and that the suppression of tumour progression and metastasis by whole-body hyperthermia may partly contribute to the prevention of angiogenesis by inhibiting VEGF production *in vivo*.

Extracellular matrix remodelling is required for endothelial–cell proliferation and migration in the process of angiogenesis, and is closely dependent on endothelial cell-derived proteinases such as MMPs (Liotta *et al*, 1991; Moses, 1997). Endothelial MMP-1 has been shown to participate in angiogenesis *in vitro* (Unemori *et al*, 1992; Fisher *et al*, 1994). A recent study by Hiraoka *et al* (1998) also reported that MT1-MMP in endothelial cells is involved in neovessel formation in mice deficient in both plasminogen activator and plasminogen. In this communication, we indicated that HT-1080 cells produced a large amount of VEGF by which the production of proMMP-1 in HUVECs was augmented, and also that heat shock effectively interfered with the production of VEGF. Furthermore, we recently reported that heat shock suppresses *in vitro* tumour invasive activity by suppressing the production of MT1-MMP and thereafter inhibiting the activation of proMMP-2 in tumour cells (Sato *et al*, 1999; Sawaji *et al*, 2000). Therefore, it is suggested that hyperthermia elicits not only an anti-angiogenic effect by inhibiting tumour-derived VEGF production but also an anti-metastatic action by suppressing the production and activation of proMMPs.

In conclusion, we demonstrated that heat shock suppresses the gene expression of three VEGF splicing variants, VEGF_121_, VEGF_165_ and VEGF_189_, and decreases the predominant product of VEGF_165_ in HT-1080 cells. In addition, the heat shock-mediated suppression of VEGF production results in the inhibition of tumour cell-induced proliferation and MMP production in endothelial cells. Furthermore, whole-body hyperthermia diminished the augmented level of VEGF in serum from patients with advanced cancers *in vivo*. Therefore, these results strongly suggest that this suppression by hyperthermia of tumour cell-derived VEGF production may explain, in part, the reason why hyperthermic therapy effectively prevents tumor growth and metastasis *in vivo*.
